# Aberrant DNA Methylation as a Biomarker and a Therapeutic Target of Cholangiocarcinoma

**DOI:** 10.3390/ijms18061111

**Published:** 2017-05-23

**Authors:** Toshiaki Nakaoka, Yoshimasa Saito, Hidetsugu Saito

**Affiliations:** Division of Pharmacotherapeutics, Keio University Faculty of Pharmacy, 1-5-30 Shibakoen, Minato-ku, Tokyo 105-8512, Japan; toshiakinakaoka1116@gmail.com (T.N.); saito-hd@pha.keio.ac.jp (H.S.)

**Keywords:** cholangiocarcinoma, DNA methylation, tumor suppressor gene, microRNA, endogenous retrovirus, anti-viral immune response

## Abstract

Cholangiocarcinoma is an epithelial malignancy arising in the region between the intrahepatic bile ducts and the ampulla of Vater at the distal end of the common bile duct. The effect of current chemotherapy regimens against cholangiocarcinoma is limited, and the prognosis of patients with cholangiocarcinoma is poor. Aberrant DNA methylation and histone modification induce silencing of tumor suppressor genes and chromosomal instability during carcinogenesis. Studies have shown that the tumor suppressor genes and microRNAs (miRNAs) including *MLH1*, *p14*, *p16*, *death-associated protein kinase* (*DAPK*), *miR-370* and *miR-376c* are frequently methylated in cholangiocarcinoma. Silencing of these tumor suppressor genes and miRNAs plays critical roles in the initiation and progression of cholangiocarcinoma. In addition, recent studies have demonstrated that DNA methylation inhibitors induce expression of endogenous retroviruses and exert the anti-tumor effect of via an anti-viral immune response. Aberrant DNA methylation of tumor suppressor genes and miRNAs could be a powerful biomarker for the diagnosis and treatment of cholangiocarcinoma. Epigenetic therapy with DNA methylation inhibitors holds considerable promise for the treatment of cholangiocarcinoma through the reactivation of tumor suppressor genes and miRNAs as well as the induction of an anti-viral immune response.

## 1. Introduction

Cholangiocarcinoma is an epithelial malignancy arising in the region between the intrahepatic bile ducts and the ampulla of Vater at the distal end of the common bile duct. The classification of cholangiocarcinoma is based on the anatomical location of tumors and includes intrahepatic, perihilar and distal cholangiocarcinoma [1]. Intrahepatic cholangiocarcinoma is defined as a cholangiocarcinoma located proximally to the second degree bile ducts in the liver. Perihilar cholangiocarcinoma is located between the second degree bile ducts and the insertion of the cystic duct into the common bile duct, whereas distal cholangiocarcinoma is located between the origin of the cystic duct and the ampulla of Vater [1]. Regarding pathological findings, most cholangiocarcinomas are well, moderately, and poorly differentiated adenocarcinomas [1].

The number of cholangiocarcinoma patients is apparently increasing worldwide. Most of cholangiocarcinomas are clinically silent, which makes early diagnosis difficult. Several imaging modalities including computed tomography (CT) and magnetic resonance imaging (MRI) as well as serological tests including serum CA19-9 concentration are helpful for the diagnosis of cholangiocarcinoma. However, these imaging modalities are not always reliable for the diagnosis of cholangiocarcinoma, and the sensitivity and specificity of these biomarkers is low [2]. The five-year survival rates are approximately 20%, as most of patients are diagnosed at an advanced stage. Although patients with cholangiocarcinoma receive chemotherapy regimens including cisplatin and gemcitabine, the effect of these chemotherapies is limited. Thus, the development of new biomarkers and therapeutic strategies against cholangiocarcinoma is urgently needed [2].

Epigenetics is an acquired modification of chromatin DNA or histone proteins such as DNA methylation and histone modification, which regulates downstream gene expression without an alteration in the DNA sequence [3]. Epigenetic alterations can be induced by aging and chronic inflammation. Aberrant DNA methylation and histone modification induce silencing of tumor suppressor genes and chromosomal instability, leading to the initiation and progression of various cancers [4,5,6,7]. MicroRNAs (miRNAs) are small non-coding RNAs that act as endogenous silencers of various target genes. miRNAs are expressed in a tissue-specific manner and play important roles in cell proliferation, apoptosis, and differentiation. We and other groups have revealed that epigenetic alterations including DNA methylation regulate not only protein-coding genes but also non-coding genes such as miRNAs in cancer cells [8,9,10,11].

Epigenetic drugs such as DNA methylation inhibitors and histone deacetylase (HDAC) inhibitors have clinical promise for cancer therapy [3,12,13]. Aberrant DNA methylation at CpG island promoters of tumor suppressor genes is frequently observed in various human malignancies including cholangiocarcinoma. The DNA methylation inhibitor 5-aza-2′-deoxycytidine (5-Aza-CdR), which is an analog of cytidine, was recently approved for the treatment of myelodysplastic syndrome (MDS). However, the effect of DNA methylation inhibitors on patients with cholangiocarcinoma remains to be elucidated. In this review, we summarize the current knowledge regarding aberrant DNA methylation of important tumor suppressor genes and miRNAs in cholangiocarcinoma as well as effects of DNA methylation inhibitors on cholangiocarcinoma.

## 2. Aberrant DNA Methylation as a Biomarker of Cholangiocarcinoma

Malignant tumors developing in the biliary tract are difficult to diagnose at an early stage because of their anatomical locations. In addition, useful biomarkers for biliary tract cancers have not been developed. Most cholangiocarcinoma patients are diagnosed at an advanced stage, and aggressive cancers easily infiltrate surrounding organs and become unresectable. The early detection of cholangiocarcinoma might improve the prognosis of patients, and the development of useful biomarkers of cholangiocarcinoma would be beneficial for prompt and more effective treatment. One of the most powerful biomarkers in cancer is DNA methylation of tumor suppressor genes. We summarized genes frequently methylated in cholangiocarcinoma in Table 1.

MLH1 protein is one component of a system of seven DNA mismatch repair (MMR) proteins that work coordinately in sequential steps to initiate the repair of DNA mismatches in humans. Several studies have demonstrated that DNA hypermethylation on the promoter region of the *hMLH1* gene is associated with a poor prognosis of patients with cholangiocarcinoma [14,15]. The *DCLK1*, *CDO1*, *ZSCAN18*, and *ZNF331* genes have been identified as novel biomarkers of colorectal cancers, and these genes are frequently methylated across gastrointestinal cancers including cholangiocarcinoma [16]. A negative correlation between promoter DNA methylation and gene expression has been observed for the *DCLK1*, *CDO1*, *ZSCAN18*, and *ZNF331* genes, suggesting that aberrant DNA methylation of these genes indicates epigenetic similarities among gastrointestinal cancers such as colon, pancreatic, and bile duct cancer. The *INK4a*-*ARF* (*CDKN2A*) locus on chromosome 9p21 encodes two tumor suppressor proteins, p16 (INK4a) and p14 (ARF), whose functions are inactivated in many human cancers. Recent studies have shown that *p16* (*INK4a*) and *p14* (*ARF*) are inactivated by DNA hypermethylation in cholangiocarcinoma, which may result in cell cycle dysregulation [17,18]. Liu et al. demonstrated that the *death-associated protein kinase* (*DAPK*) gene is suppressed by promoter hypermethylation in cholangiocarcinoma. Silencing of the *DAPK* gene by DNA hypermethylation results in resistance to apoptosis and immunological surveillance [22]. In addition, it has been reported that *p53* mutation combined with DNA methylation of the *DAPK*, *p14* (*ARF*), and *ASC* genes correlates with malignancy and poor prognosis of patients with cholangiocarcinoma [19].

Cancer cells are considered to be heterogeneous with a hierarchy of “stemness” in solid cancer tissues. Stem cells have the ability to self-renew and to generate mature cells of various tissues through differentiation. Cancer stem cells, a subpopulation of cancer cells with distinct stem-like properties, is responsible for tumor initiation, invasive growth, and metastasis formation [26]. As cancer stem cells are resistant to conventional chemotherapies and radiation therapy, it would be desirable to develop a therapeutic strategy specifically targeting cancer stem cells. Sriraksa et al. reported that hypermethylation of multiple CpG sites of genes associated with a stem cell-like phenotype is a common molecular aberration in cholangiocarcinoma [27], indicating that aberrant DNA methylation plays a critical role in “cancer stemness” of cholangiocarcinoma.

Early diagnosis is very important for patients with refractory cancers, but detection of cholangiocarcinoma at an early stage is still challenging because it is difficult to visualize biliary tract tumors by existing imaging modalities [28]. In order to overcome this problem, Shin et al. developed a useful method for the detection of cholangiocarcinoma cells using bile fluid [23]. This method involving DNA methylation assay consisting of a five-gene panel (*CCND2*, *CDH13*, *GRIN2B*, *RUNX3* and *TWIST1*) is able to detect cholangiocarcinoma cells with a sensitivity of 83% and a specificity of 100%. Less invasive examinations such as this method using bile fluid are important for minimizing the burden on the patient. These studies have shown that detection of DNA methylation is a powerful diagnostic strategy for patients with cholangiocarcinoma.

## 3. DNA Methylation Inhibitors Are Promising Therapeutic Agents against Cholangiocarcinoma

**Figure 1:**
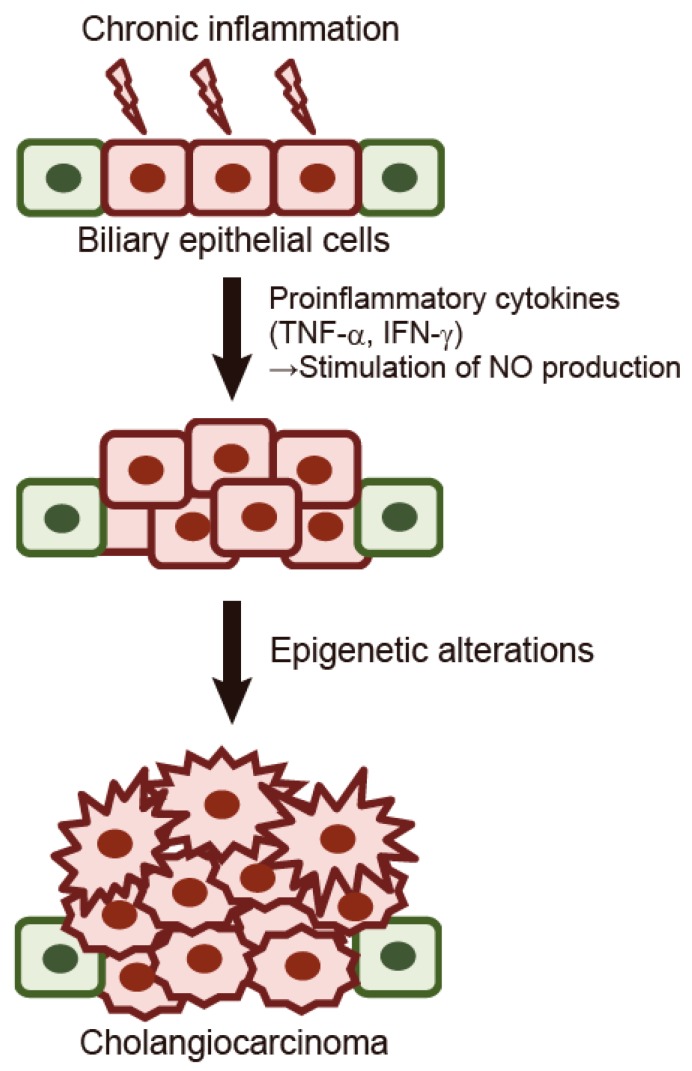
The molecular mechanism underlying the initiation of cholangiocarcinoma. When chronic inflammation and cholestasis arise due to liver injury, proinflammatory cytokines such as tumor necrosis factor-α (TNF-α) and interferon-γ (IFN-γ) stimulate the biliary epithelium to generate nitric oxide (NO). NO alters epigenetic regulation including DNA methylation, which leads to accelerated growth of biliary epithelial cells. Accelerated proliferation of biliary epithelial cells promotes aberrant DNA methylation of tumor suppressor genes, leading to the initiation of cholangiocarcinoma. Green cells, normal biliary epithelial cells; Red cells, precancerous biliary epithelial cells; Jagged-shaped red cells, cholangiocarcinoma cells.

Chronic inflammation in the liver may contribute to malignant transformation of cholangiocytes [29]. It is assumed that persistent inflammation promotes carcinogenesis through DNA damage and tissue repair as well as activation of cytokines and other growth factors [30]. A previous study demonstrated that cholangiocyte-derived cytokines, such as interleukin 6 (IL-6), transforming growth factor-α (TGF-α), and tumor necrosis factor-α (TNF-α) regulate cholangiocyte intracellular signaling and promote carcinogenesis [31]. Spirli et al. demonstrated that proinflammatory cytokines such as TNF-α and interferon (IFN)-γ stimulate the biliary epithelium to generate nitric oxide (NO) via induction of nitric oxide synthase 2 (NOS2) [32]. They also showed that NOS2 expression is significantly increased in the biliary epithelium of patients with primary sclerosing cholangitis (PSC) [32]. Moreover, recent studies have shown that NO directly and indirectly affects numerous epigenetic mechanisms. NO has been shown to alter DNA methylation and histone modifications by altering epigenetic enzymes [33]. Figure 1 shows the molecular mechanism underlying the initiation and progression of cholangiocarcinoma. When chronic inflammation and cholestasis arise due to liver injury, proinflammatory cytokines such as TNF-α and IFN-γ stimulate the biliary epithelium to generate NO. NO alters epigenetic regulation including DNA methylation, which leads to accelerated growth of biliary epithelial cells. Accelerated proliferation of biliary epithelial cells promotes aberrant DNA methylation of tumor suppressor genes, leading to the initiation of cholangiocarcinoma. Wehbe et al. previously reported that IL-6 contributes to the growth of cholangiocarcinoma cells through aberrant DNA methylation on the promoter region of tumor suppressor genes [24]. IL-6 decreased DNA methylation level on the promoter region of the *EGFR* gene, which leads to the increased expression of the EGFR protein. These findings suggest that persistent cytokine stimulation in biliary epithelial cells could promote the initiation and progression of tumors via epigenetic alterations. Wang et al. showed that suppression of the tumor suppressor *liver kinase B1* (*LKB1*) due to aberrant DNA methylation is associated with enhanced Wnt signaling and malignant characteristics of human cholangiocarcinoma [25]. The expression of the *LKB1* gene was suppressed in cholangiocarcinoma tissues relative to adjacent normal tissues and knockdown of *LKB1* enhanced the growth, migration, and invasion of tumors, along with the activation of Wnt signaling.

**Figure 2:**
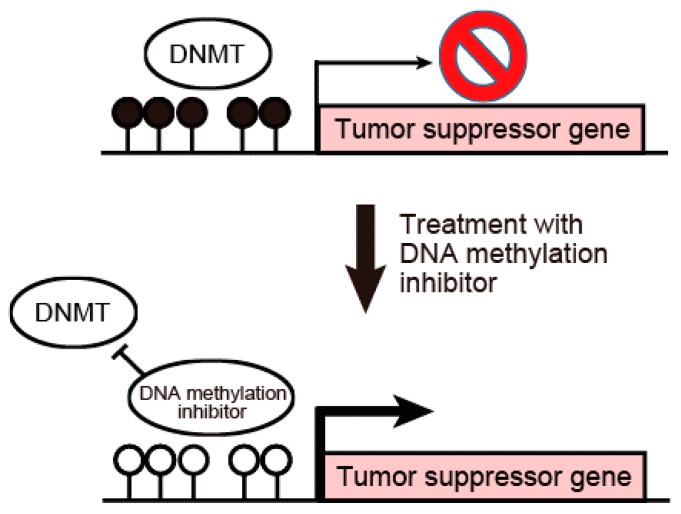
Activation of tumor suppressor genes by the inhibition of DNA methylation on their promoter regions. In cancer cells, tumor suppressor genes are silenced by DNA hypermethylation on CpG island promoter regions. DNA methylation inhibitors such as 5-Aza-CdR can reactivate epigenetically silenced tumor suppressor genes by the inhibition of DNA methylation on promoter regions. Solid circle, methylated DNA; clear circle, unmethylated DNA.

Figure 2 shows a scheme for the activation of tumor suppressor genes by the inhibition of DNA methylation on their promoter regions. In cancer cells, tumor suppressor genes are silenced by DNA hypermethylation on CpG island promoter regions. DNA methylation inhibitors such as 5-Aza-CdR can reactivate epigenetically silenced tumor suppressor genes by the inhibition of DNA methylation on promoter regions. Several studies have evaluated the effect of DNA methylation inhibitors on cholangiocarcinoma. The DNA methylation inhibitor zebularine inhibited human cholangiocarcinoma cells through the alteration of DNA methylation status [34]. Zebularine exerted an anti-tumor effect on cholangiocarcinoma cells through the suppression of DNA methyltransferases. Zebularine altered the DNA methylation status and suppressed the Wnt signaling pathway, resulting in the decreased expression of CTNNB1. Several reports have indicated that tumor suppressor genes that were silenced in cholangiocarcinoma could be reactivated by the DNA methylation inhibitor 5-Aza-CdR [20,35]. Liu et al. reported that treatment of cholangiocarcinoma cells with 5-Aza-CdR inhibited cell growth and induced apoptosis by the reactivation of p53-BAX mitochondrial apoptosis genes [20]. Xiang et al. demonstrated that knockdown of the major DNA methyltransferase *DNMT1* restores the expression levels of tumor suppressor genes, which results in the inhibition of the proliferation of cholangiocarcinoma cells [21]. These findings suggest that various tumor suppressor genes are inhibited by DNMT1-induced DNA hypermethylation in their promoter regions, which enhances the proliferation, migration and invasion of cholangiocarcinoma cells. The biological effects of tumor suppressor genes frequently methylated in cholangiocarcinoma are summarized in Table 1. DNA methylation inhibitors such as 5-Aza-CdR and zebularine might have great promise for the treatment of cholangiocarcinoma. However, these DNA methylation inhibitors affect without gene specificity. Lee et al. showed that human N-α-acetyltransferase 10 protein (hNaa10p) contributes to tumorigenesis by facilitating DNMT1-mediated tumor suppressor gene silencing [36]. They confirmed that the oncogenic potential of hNaa10p depends on its interaction with DNMT1. hNaa10p positively regulates DNMT1 enzymatic activity by facilitating its binding to DNA and recruitment to the promoters of tumor suppressor genes such as E-cadherin. These data suggest that DNMT1-induced gene silencing may affect tumor suppressor genes rather than oncogenes in cancer cells. Further studies are necessary to develop DNA methylation inhibitors that specifically affect only the CpG island promoter region of tumor suppressor genes to reduce the side effects of epigenetic therapy.

## 4. Suppression of Tumor Suppressor miRNAs by DNA Methylation in Cholangiocarcinoma

The deregulation of miRNAs induces the initiation and progression of cancers by modifying their target tumor suppressor genes or oncogenes [37]. Braconi et al. showed that IL-6 can regulate the activity of DNMT1 by miRNAs in cholangiocarcinoma cells [38]. They verified that *miR-148a* and *miR-152* regulate DNMT1 expression as their targets. They also showed that IL-6 can regulate the activity of DNMT1 and expression of DNA methylation-dependent tumor suppressor genes by modulation of *miR-148a* and *miR-152*. These findings provide a link between this inflammation-associated cytokine and oncogenesis in cholangiocarcinoma. In addition, several studies have shown that tumor suppressor miRNAs are regulated by DNA methylation. Meng et al. reported that the expression of DNA methyltransferases was increased by IL-6 overexpression and the tumor suppressor *miR-370* was inactivated by DNA methylation in cholangiocarcinoma cells [39]. The oncogene *mitogen-activated protein kinase kinase kinase 8* (*MAP3K8*) was identified as a target of *miR-370*. 5-Aza-CdR increased the expression of *miR-370* in malignant cells, while the expression in non-malignant cells was unchanged. Thus, IL-6 may contribute to tumor growth by the modulation of *miR-370* expression in cholangiocarcinoma cells. These findings define a mechanism by which inflammation-associated cytokines can epigenetically modulate gene expression and contribute to the initiation and development of cholangiocarcinoma.

Iwaki et al. also showed that *miR-376c* was regulated by DNA methylation and associated with tumor suppression by targeting *growth factor receptor-bound protein 2* (*GRB2*) [40]. They found higher methylation levels of CpG sites upstream of the *miR-376c* gene in cholangiocarcinoma cells relative to normal intrahepatic biliary epithelial cells. The direct target genes and biological functions of miRNAs frequently methylated in cholangiocarcinoma are summarized in Table 2. Since miRNAs regulate several target genes including cancer-related genes, the replacement of tumor suppressor miRNAs might have implications for the treatment of cholangiocarcinoma as well as the activation of tumor suppressor miRNAs by epigenetic therapy using chromatin-modifying agents.

## 5. Therapeutic Perspectives of DNA Methylation Inhibitors against Cholangiocarcinoma

Other anti-tumor effects of chromatin-modifying drugs have been demonstrated in cancers including colon cancer. One of these other anti-tumor effects is the induction of tumor cell differentiation. Hatano et al. previously showed that DNA demethylation exerts a tumor-suppressive effect on colon cancers by inducing tumor differentiation [41]. They found that the promoter region of the *Caudal type homeobox 1* (*CDX1*) gene was methylated specifically in colon cancer cells. The upregulation of *CDX1* increased the expression of genes related to intestinal differentiation. This suggested that the promoters of transcriptional factor genes regulating cell differentiation were silenced by DNA hypermethylation in colon cancer cells to sustain their undifferentiated status.

**Figure 3:**
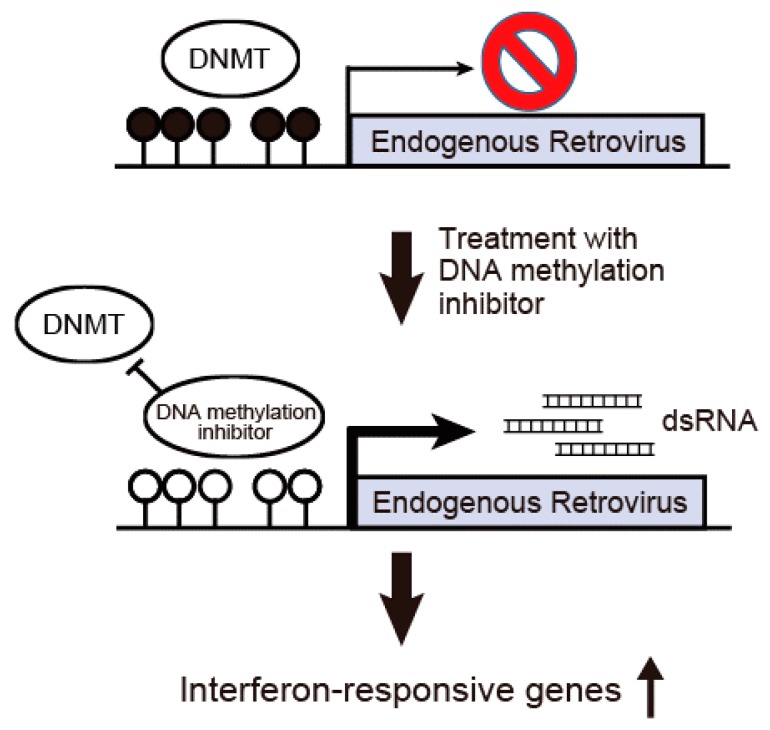
Activation of an anti-viral immune response induced by the inhibition of DNA methylation. In a normal state, the 5′ LTR sequences of endogenous retrovirus (ERVs) are heavily methylated and the expression of ERVs is silenced. When DNA methylation at the 5′ LTR sequences is inhibited by DNA methylation inhibitors, the expression of ERVs is induced. Increased expression of dsRNAs derived from ERVs leads to the induction of an anti-viral immune response such as the activation of interferon-responsive genes.

Recent studies have proved that the major effect of DNA methylation inhibitors is to induce interferon-responsive genes by increasing double-stranded RNA (dsRNA) containing endogenous retrovirus (ERV) [42,43]. Different ERV gene families constitute about 8% of the human genome and are considered to be long terminal repeat [44] retrotransposons. Innate immune responses are activated by the expression of ERV-producing nucleic acids or proteins with viral signatures [45]. Roulois et al. recently proposed that 5-Aza-CdR could be used to target colorectal cancer stem cells by inducing viral mimicry [42]. Their data suggested that the induction of dsRNAs is derived at least in part from ERV elements, which activate the MDA5/MAVS RNA recognition pathway. Figure 3 shows a scheme for the activation of an anti-viral immune response induced by the inhibition of DNA methylation. In a normal state, the 5′ long terminal repeat (LTR) sequences of ERVs are heavily methylated and the expression of ERVs is silenced. When DNA methylation at the 5′ LTR sequences is inhibited by DNA methylation inhibitors, the expression of ERVs is induced. Increased expression of dsRNAs derived from ERVs leads to the induction of an anti-viral immune response such as the activation of interferon-responsive genes.

We also reported that DNA methylation inhibition suppresses intestinal tumor organoids by inducing anti-viral response [46]. We established tumor organoids derived from the *Apc*^min/+^ mouse, a model of colon cancer, using a new 3D culture system that allows Lgr5-positive stem cells to form cyst-like structures (organoids) [47]. This organoid culture system closely recapitulates the properties of the original tumors, and is useful for drug screening and precision medicine [48]. We demonstrated that 5-Aza-CdR shrinks intestinal tumor organoids derived from *Apc*^min/+^ mice [46]. We revealed that the expression of interferon-responsive genes such as *Irf7*, *Rig1* and *Mda5* was increased by DNA methylation inhibition in tumor organoids after 5-Aza-CdR treatment or *Dnmt1* knockdown. The expression of murine ERVs was significantly upregulated after the treatment of tumor organoids with 5-Aza-CdR. These findings suggested that treatment with DNA methylation inhibitors to activate an innate immune response would be beneficial for patients with various types of cancers including cholangiocarcinoma. Wrangle et al. showed that DNA methylation inhibitors can upregulate transcripts and protein of PD-L1, a key ligand mediator of immune tolerance [49]. Through analysis of samples from The Cancer Genome Atlas (TCGA), they also demonstrated that a significant proportion of primary non-small cell lung cancers (NSCLCs) have a low expression of DNA methylation inhibitor-induced immune genes such as PD-L1. Their data suggested that a combination of chromatin-modifying agents with immune checkpoint blockade therapies would activate the immune response of the host to cancer cells.

The development of anti-metabolite drugs that are dependent on the cell cycle of cancer cells has revealed a serious problem in that they also act on normal cells and normal stem cells. Therefore, molecular targeting therapeutic agents have been developed to avoid seriously damaging normal cells. One such molecular targeting therapeutic agent is herceptin, approved for the treatment of breast cancer. Although herceptin has improved the relapse-free survival of patients with breast cancer [50], it is still very difficult to eliminate the cancer completely, because cancers have various mutations and different forms of aberrant epigenetic status. In this respect, chromatin-modifying drugs have great promise for cancer therapy because the modification of epigenetic status alone can inhibit various tumor characteristics such as proliferation, migration, invasion, and dedifferentiation. It has been demonstrated that reprofiling of food and drug administration (FDA)-approved drugs in combination with chromatin-modifying drugs can be implemented into clinical trials for colon cancer [51].

In conclusion, aberrant DNA methylation of tumor suppressor genes and miRNAs could be a powerful biomarker for the diagnosis and treatment of cholangiocarcinoma. Epigenetic therapy with DNA methylation inhibitors hold considerable promise for the treatment of cholangiocarcinoma through the reactivation of tumor suppressor genes and miRNAs as well as the induction of an anti-viral immune response.

## Figures and Tables

**Table 1:** Genes frequently methylated in cholangiocarcinoma.

Gene	Function	Sample	Reference
*MLH1*	DNA repair	tissue	[14,15]
*DCLK1*	stemness	tissue	[16]
*CDO1*	growth	tissue	[16]
*ZSCAN18*	unknown	tissue	[16]
*ZNF331*	growth invasion	tissue	[16]
*p14* (*ARF*)	cell cycle regulator	tissue	[17,18,19]
*p16* (*INK4a*, *CDKN2A*)	cell cycle regulator	tissue QBC939 cell line	[17,18,19,20,21]
*DAPK*	apoptosis	tissue QBC939 cell line	[19,20,22]
*CCND2*	growth	bile fluid	[23]
*CDH13*	growth invasion	bile fluid	[23]
*GRIN2B*	growth	bile fluid	[23]
*RUNX3*	growth differentiation	bile fluid	[23]
*TWIST1*	migration invasion	bile fluid	[23]
*EGFR*	growth	Mz-ChA-1 cell line	[24]
*LKB1*	growth migration invasion	tissue HuH-28 cell line RBE cell line SSP-25 cell line	[25]

**Table 2:** miRNAs frequently methylated in cholangiocarcinoma.

miRNA	Target Gene	Function	Sample	Reference
*miR-370*	*MAP3K8*	cell proliferation	MzChA-1 cell line KMCH-1 cell line	[39]
*miR-376c*	*GRB2*	migration	HuCCT1 cell line	[40]
